# The Influence of Pressure on the Intrinsic Dissolution Rate of Amorphous Indomethacin

**DOI:** 10.3390/pharmaceutics6030481

**Published:** 2014-08-20

**Authors:** Korbinian Löbmann, Konstantina Flouda, Danwen Qiu, Theodosia Tsolakou, Wenbo Wang, Thomas Rades

**Affiliations:** Department of Pharmacy, University of Copenhagen, Copenhagen 2100, Denmark; E-Mails: kon.flouda@gmail.com (K.F.); danwenqiu@gmail.com (D.Q.); teotsolakou@gmail.com (T.T.); wwbsgdtc@126.com (W.W.); thomas.rades@sund.ku.dk (T.R.)

**Keywords:** amorphous, crystalline, dissolution, compression pressure, indomethacin

## Abstract

New drug candidates increasingly tend to be poorly water soluble. One approach to increase their solubility is to convert the crystalline form of a drug into the amorphous form. Intrinsic dissolution testing is an efficient standard method to determine the intrinsic dissolution rate (IDR) of a drug and to test the potential dissolution advantage of the amorphous form. However, neither the United States Pharmacopeia (USP) nor the European Pharmacopeia (Ph.Eur) state specific limitations for the compression pressure in order to obtain compacts for the IDR determination. In this study, the influence of different compression pressures on the IDR was determined from powder compacts of amorphous (ball-milling) indomethacin (IND), a glass solution of IND and poly(vinylpyrrolidone) (PVP) and crystalline IND. Solid state properties were analyzed with X-ray powder diffraction (XRPD) and the final compacts were visually observed to study the effects of compaction pressure on their surface properties. It was found that there is no significant correlation between IDR and compression pressure for crystalline IND and IND–PVP. This was in line with the observation of similar surface properties of the compacts. However, compression pressure had an impact on the IDR of pure amorphous IND compacts. Above a critical compression pressure, amorphous particles sintered to form a single compact with dissolution properties similar to quench-cooled disc and crystalline IND compacts. In such a case, the apparent dissolution advantage of the amorphous form might be underestimated. It is thus suggested that for a reasonable interpretation of the IDR, surface properties of the different analyzed samples should be investigated and for amorphous samples the IDR should be measured also as a function of the compression pressure used to prepare the solid sample for IDR testing.

## 1. Introduction

In tablet manufacturing, direct compression is an advanced technique for the development of many drugs into solid dosage forms and its formulation and process variables play a crucial role on the product’s quality [1]. Dissolution rate and solubility of a new drug candidate are crucial factors that influence a drugs’ tendency to supersaturate and thus, its bioavailability. Therefore, an understanding of dissolution behavior is necessary during the developmental stage. In previous studies, the effect of compression pressure on dissolution rate of solid materials has been reported. Iranloye* et al*. suggested that compression pressure had no influence on the dissolution rate of compressed disks of crystalline salicylic acid, aspirin and mixtures of aspirin and salicylic acid [2]. In addition, Velasco* et al*. reported that there is no relationship between compression pressure and the dissolution profiles of diclofenac sodium from matrix tablets [3]. A common situation during the compression of crystalline material is that the crystallites of the compound will orientate less randomly (texturization). Tenho* et al*. suggested that although the preferred orientation of crystallites (texture) has a very weak correlation with the intrinsic dissolution rate of some active pharmaceutical ingredients, it must be considered during accurate dissolution studies. However, the effect of texture might be obscured when amorphicity is present in the sample [4].

Within the pharmaceutical industry, new drug candidates tend to be poorly water soluble [5] and consequently present a low dissolution rate [6,7]. Strategies to overcome poor aqueous solubility and enhance the dissolution rate of solid drugs include amorphization, formation of co-crystals, and the use of metastable crystalline polymorphs or solid drug polymer dispersions [8,9]. Amorphization leads to the amorphous form of a drug which, compared to the crystalline form, can be defined by randomly arranged molecules and lack of the three dimensional position and orientation long-range order [10]. Thus, the amorphous form is a high-energy state that has a higher free energy than the thermodynamically stable crystalline form. As a result, the amorphous form frequently increases solubility and dissolution rate [11], while maintaining the pharmacological activity of the substance [12]. Several methods can be applied to obtain the amorphous form among which spray drying, freeze drying, quench cooling, and vibrational ball milling are the most common. It is known that the production method as well as process parameters can influence the dissolution profile of the amorphous form [11,13].

For amorphous systems the long-term physical stability is crucial for their benefits in product development [14]. However, amorphous systems are less stable than their crystalline counterparts and tend to transfer back to their crystalline structure. Crystallization often is associated with the molecular mobility of the amorphous matrix [15,16] and therefore, problems can occur during manufacturing and storage. Storage conditions such as humidity and temperature have been investigated extensively, nevertheless, there is a limited understanding on the impact of manufacturing processes such as compression on the stability of amorphous solids [17]. Reported changes in stability varied depending on the compressed systems, for instance compression pressure enhanced the physical stability of spray-dried Naproxen/PVP–VA 64 [18] while compression increased the crystallization of melt-cooled indomethacin [19].

Even if the drug remains amorphous after manufacturing and storage, there is still a possibility of recrystallization during dissolution (e.g., after administration) and therefore, the expected bioavailability enhancement from using an amorphous form, can be reduced [20]. In previous studies, the effect of compression pressure on the dissolution of amorphous compounds was shown to be minor. Nanjwade* et al*. reported similar dissolution profiles for low and high direct compressed amorphous cefuroxime axetil disks [21]. In addition, Wlodarski* et al*. suggested that with increasing compression pressure, there are insignificant differences between the dissolution rates of ball milled tadafil compacts [22].

As a routine method in preformulation,* in vitro* dissolution experiments are commonly applied to determine potential advantages of an amorphous formulation approach. In order to minimize particle size effects, intrinsic dissolution is usually applied to obtain the intrinsic dissolution rate (IDR) for a given compound or its formulation as the area of the dissolving compact theoretically remains constant [23]. Traditional methods to determine the IDR of a compound are available in the USP (rotating disc and stationary disc system). However, neither the USP nor the Ph.Eur state specific limitations for the compression pressure during disc formation. Due to the absence of these limitations, the aim of this study was to investigate whether different compression pressures influenced the IDR of amorphous IND and a glass solution of IND and poly(vinylpyrrolidone) (PVP). Because of a different compaction behavior of the amorphous form compared to the crystalline form of indomethacin, the amorphous form is easier to compress and may show a higher dependency of the IDR on the compression pressure.

## 2. Materials and Methods

### 2.1. Materials

Crystalline γ-indomethacin (IND, *M*_r_ = 357.79 g/mol) was purchased from Hawkins Inc. Pharmaceutical Group (Minneapolis, MN, USA). Poly(vinylpyrrolidone) K30 (PVP, *M*_r_ = 40,000 g/mol) was obtained from Unikem (Copenhagen, Denmark). The materials were used as received.

### 2.2. Methods

#### 2.2.1. Preparation of Amorphous Indomethacin (IND)

Amorphous IND and its respective glass solution with PVP (IND–PVP) were obtained by vibrational ball milling (Mixer Mill MM400, Retch GmbH and Co, Haan, Germany) at 30 Hz in a cold room at 6 °C. Five hundred milligrams of bulk IND and 1000 mg of IND–PVP mixture (1:1 *w*/*w*) were placed into 25 mL milling jars together with two stainless steel balls (12 mm). The milling time was set to 90 and 60 min, respectively. For comparison, amorphous IND was also prepared by quench cooling. A total of 1000 mg crystalline IND was melted, in a stainless steel beaker placed on a hot plate (approximately 170 °C), and subsequently poured into the stainless steel cylinders for intrinsic dissolution testing. The cylinders were mounted on a flat stainless steel surface at room temperature to ensure a flat surface after the pouring of the melt and its quenching (quenched-in IND). In all experiments only freshly prepared samples were used for the experiments.

#### 2.2.2. X-ray Powder Diffraction (XRPD)

X-ray powder diffraction analysis (XRPD) was carried out to evaluate the solid state of the powders and the discs. XRPD was performed using an X’Pert PANalytical X’Pert PRO X-ray diffractometer (PANalytical Almelo, The Netherlands, Cu*K*α anode; λ = 1.54187 Å). An acceleration voltage and current of 45 kV and 40 mA were used. The samples were scanned over the range 5°–35° 2θ with a step size of 0.0262606° and scan speed of 0.067335° 2θ/s. The data were collected and analysed by X’Pert Data Collector software (PANalytical B.V., Almelo, The Netherlands).

#### 2.2.3. Intrinsic Dissolution Studies

##### Dissolution Procedure

The intrinsic dissolution rate (IDR) was determined from powder compacts, compressed at different compression pressures, using a laboratory press (Graseby Specac Hydraulic Press, Specac, Orpington, UK). Compacts of IND–PVP, amorphous IND and crystalline IND (all 100 ± 0.02 mg) were directly compacted for 1 min into the stainless steel cylinder, resulting in discs at one side of the cylinder with flat surface and a diameter of 1 cm (constant surface area of 0.785 cm^2^). In order to prevent sticking of compacts to die, the tablet punch was pre-treated with magnesium stearate as lubricant and anti-adherent. Amorphous IND was compressed at 37, 125, 187, 219, 250 and 312 MPa, IND–PVP at 25, 94, 375 and 625 MPa, and crystalline IND at 63 and 125 MPa. The final compacts were visually observed to study the effect of compaction pressure on their surface properties. In addition, the IDR of the directly quenched-in IND discs was determined and compared to the milled amorphous IND compacts.

For the dissolution test an Erweka DT70 rotating disc dissolution apparatus (Erweka GmbH, Heusenstamm, Germany) was used. The holders with the sample compacts were placed in vessels with 900 mL of 0.01 M phosphate buffer (pH 7.3, 37 ± 0.5 °C) as dissolution medium. The samples were rotated at 50 rpm, and 5 mL aliquots of test medium were withdrawn at predetermined time points (1, 2, 4, 6, 8, 10, 15 and 20 min). The withdrawn medium was replaced by 5 mL of preheated buffer. Samples were analyzed for indomethacin concentration using a UV–vis spectrophotometer and for quantification, a standard curve was prepared using six different concentrations of indomethacin (see below). All measurements were carried out in triplicate. The dissolution rates were estimated by linear fitting of the dissolution curves. The IDR was calculated as drug release per accessible area (mg cm^−2^).

##### UV Analysis

A UV–vis spectrophotometer (UV-1601, Shimadzu, Kyoto, Japan) was used to determine the concentration and therefore the amount of the dissolved IND in the samples taken during the dissolution. Reference spectra were recorded for the buffer and the buffer-PVP solutions in a range from 200 to 500 nm, in order to confirm no absorbance and interference with the drug absorption in this specific range. Three hundred and twenty nanometers was selected as the wavelength for IND detection in all measurements. Standard solutions (4.5, 3.4, 2.6, 1.4, 0.5 and 0.14 μg/mL) for IND were prepared by dissolving the drug in phosphate buffer (0.01 M, pH 7.3) and making the appropriate dilutions. The resulting standard curve was linear in the concentration range (*R*^2^ = 0.9999). To examine if there is an effect on the absorbance of IND by the addition of PVP, standard solutions of IND–PVP (ratio 1:1) in buffer were measured and no deviation occurred from the standard solutions of pure IND dissolved in buffer.

##### Data Analysis

A single factor ANOVA for the dissolved amount at each time point was performed to observe differences in the dissolution behavior of the compacts. Moreover, the dissolution rates (slope of the fitted line through the data points) for IND–PVP and pure IND compacts were compared by linear regression to see whether the slopes are different. In both cases the differences were considered significant if the *p*-value was lower than 0.05 (95% confidence level).

## 3. Results and Discussion

### 3.1. Solid State Analysis

**Figure 1:**
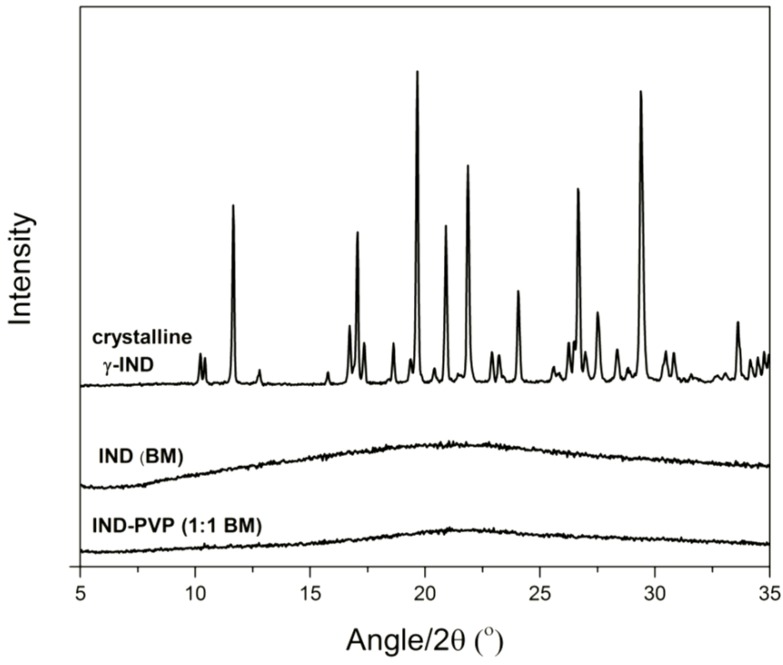
X-ray powder diffraction (XRPD) diffractograms of crystalline starting material (crystalline γ-IND), amorphous indomethacin (IND) and IND–poly(vinylpyrrolidone) (PVP) glass solution (1:1 *w*/*w*) after ball milling (BM).

The starting material, crystalline γ-IND, and the ball milled IND samples were analyzed with XRPD. Figure 1 shows the XRPD patterns of the crystalline γ-IND, freshly ball milled IND, and the glass solution of IND–PVP. It can be seen that IND can be converted into its amorphous form upon ball milling, either as a pure drug or as a glass solution with PVP. As reported previously, the presence of an amorphous form is indicated by the absence of diffraction peaks and the appearance of a halo pattern [24,25,26].

**Figure 2:**
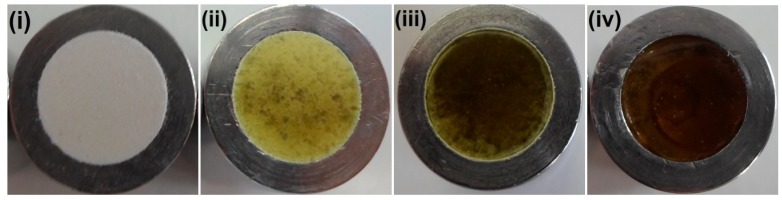
Macroscopic images after compaction: (**i**) crystalline IND (125 MPa); (**ii**) amorphous IND (187 MPa); (**iii**) amorphous IND (312 MPa) and (**iv**) quenched-in IND.

During the compression experiments, it was observed that there was a critical compression pressure of >63, >37 and >25 MPa, for crystalline IND, amorphous IND and IND–PVP, respectively, that had to be applied to obtain stable compacts. In the case of crystalline IND there was also an upper limit of >125 MPa above which the compacts broke. After compaction and before the dissolution test, macroscopic evaluation was performed on the compacts in order to analyze the surface properties upon the different compression pressure. Generally, for IND–PVP compacts no significant differences were observed in their surface appearance,* i*.*e*., all appeared as powder compacts. Similarities in surface properties were observed for the two crystalline IND compacts as well (Figure 2i). In contrast, pure amorphous IND compacts, presented differences with increasing compression pressure. At low compression pressure the compacts appeared similar to those of IND–PVP,* i*.*e*., simple powder compacts (Figure 2ii). Above a compression pressure of 250 MPa, however, the compact was clear, glassy and homogeneous (Figure 2iii), where the single particles sintered to form a smooth surface. The latter disc compacts were of similar appearance as the directly quenched-in IND discs (Figure 2iv).

### 3.2. Intrinsic Dissolution Studies

**Figure 3:**
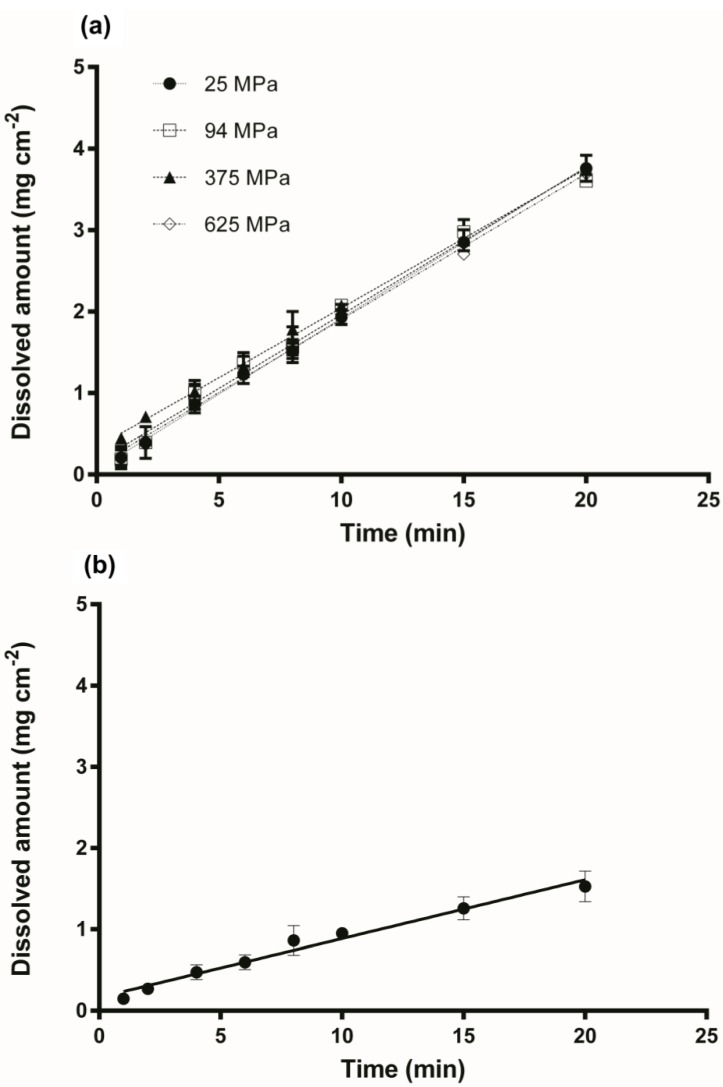
(**a**) Dissolution profiles of IND–PVP compacts, prepared by different compression pressure (25, 94, 375 and 625 MPa); (**b**) Dissolution profile of crystalline IND compacts compressed at 125 MPa (dissolution experiments were carried out at 37 ± 0.5 °C for 20 min).

The intrinsic dissolution studies were performed under stable conditions (constant surface area, temperature, agitation, pH and ionic strength), in order to investigate the dissolution profiles of the amorphous IND compacts, prepared by applying different compression pressures. The dissolved amount of IND per unit area as a function of time is illustrated in the IDR profiles of the different compacts in Figure 3 and Figure 5. Although crystalline IND compacts presented similarities in their surface, those compressed at 63 MPa were not stable enough for the duration of the dissolution test. Thus, only the dissolution profile of the compacts compressed at 125 MPa was used for the analysis (Figure 3b).

**Figure 4:**
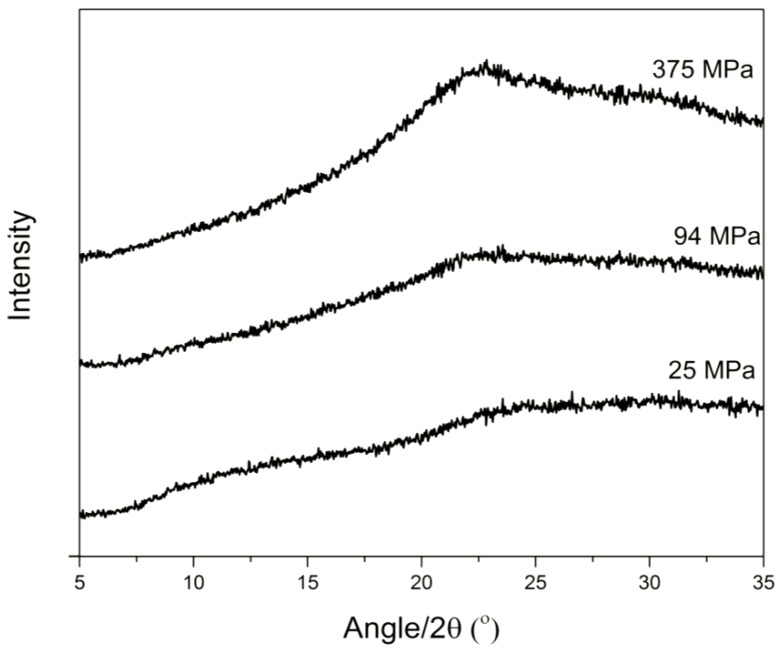
XRPD diffractograms for IND–PVP compacts after the dissolution test. The appearance of the halo confirms that the compacts are still amorphous after the dissolution test.

In line with the visual observation of the disc surfaces, the different IND–PVP compacts presented almost the same intrinsic dissolution profile, suggesting that the compression pressure had no detectable influence on the IDR on these samples (Figure 3a). The ANOVA analysis for the IND–PVP compacts showed that there was no statistical difference between the data for the individual time points. Moreover, linear regression showed that all the slopes were not significantly different (*p* = 0.1025). After the dissolution experiments, the surfaces of the IND–PVP compacts appeared glossy. It is suggested that the presence of PVP is forming a small gel like layer on the surface of the compact upon contact with the dissolution medium. The formation of such a PVP gel layer may be the reason for the similar dissolution rate, as this layer then represents the diffusion layer for the drug. Thus, the rate limiting step for the dissolution might be the diffusion of the drug through the gel layer rather than the influence of the compaction pressure [27]. However, the presence of PVP in the compacts, enhanced the dissolution properties of the drug [28] compared to the crystalline form and furthermore, prevented the drug from recrystallization, as no diffraction peaks were observed by XRPD from the compacts after dissolution (Figure 4). This confirms the stabilizing properties of the polymer (PVP) on the amorphous form of IND within the solid dispersion [29,30].

**Figure 5:**
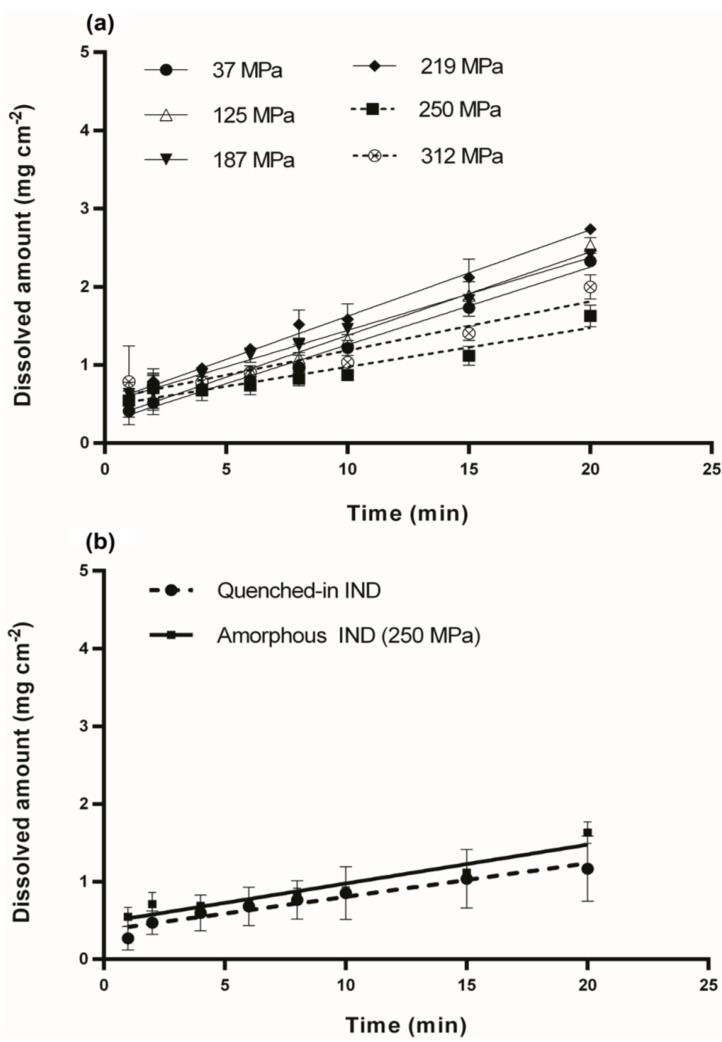
(**a**) Dissolution profiles of amorphous IND compacts, prepared by different compression pressure (37, 125, 187, 219, 250 and 312 MPa); (**b**) Dissolution profiles of quench-in IND discs and milled IND compacts compressed at 250 MPa (dissolution experiments were carried out at 37 ± 0.5 °C for 20 min).

On the other hand, the IDR of the amorphous IND compacts showed differences as a function of the applied compaction pressure (Figure 5a). As the compression pressure was increased from 37 to 125 MPa the IDR remained almost the same. Linear regression showed that the slopes were not significantly different (*p* = 0.2). Above a critical compression pressure of 250 MPa, however, the IDR was approximately half compared to the samples compressed below that pressure. This can be explained by the altered surface,* i*.*e*., sintered particles, of the compact at compression pressures of 250 MPa and higher. In order to investigate this further, the IDR of a homogeneous clear and glassy solid prepared by direct quenching of the melt of IND in the sample holder was prepared, and it was found that the IDR of the quenched-in IND was similar to the IDR for amorphous IND compacts compressed at 250 MPa and above. Figure 5b illustrates the dissolution profile of the quenched-in IND discs compared to the profile of the milled IND compact compressed at 250 MPa. Linear regression showed that the slopes were not significantly different (*p* = 0.3934).

In comparison to the powder compact, the sintered compact appears as one large fused particle with constant surface area. It is suggested that the powder compacts at lower compaction pressure have a lower surface stability upon the dissolution process,* i*.*e*., single particles dissolve, leaving holes within the disc surface, thus increasing the specific surface area of the disc and at the same time the dissolution rate of the amorphous IND compacts.

**Figure 6:**
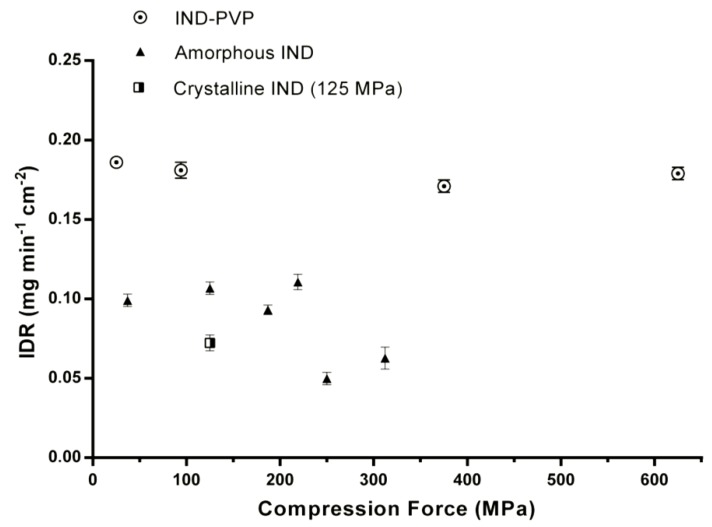
IDRs of IND–PVP, amorphous (milled) IND and crystalline IND compacts. The IDRs are depicted as a function of the applied compression pressure.

Table 1 summarizes the IDR of all samples after the dissolution studies. Moreover, for a better visualization, the IDRs of IND–PVP as well as the pure amorphous IND compacts are depicted as a function of the compression pressure in Figure 6. It can be seen that in the IND–PVP glass solution, the compression pressure had no influence on the dissolution behavior of the compacts. As expected, the IND–PVP compacts also had the highest intrinsic dissolution rate compared to the amorphous and crystalline IND. In contrast, the dissolution behavior of pure amorphous compacts is influenced by the applied compression pressure and is decreasing above a critical value (250 MPa).

**Table 1:** IDR determined from the slopes of the dissolution profiles for IND–PVP, amorphous IND and crystalline IND compact as well as from the quenched-in IND discs.

Sample	Compression Pressure (MPa)	IDR (mg min^−1^ cm^−2^)
IND–PVP	25	0.186 ± 0.003
	94	0.181 ± 0.005
	375	0.171 ± 0.004
	625	0.179 ± 0.004
Amorphous IND (milled)	37	0.099 ± 0.004
	125	0.107 ± 0.004
	187	0.093 ± 0.003
	219	0.111 ± 0.005
	250	0.049 ± 0.006
	312	0.062 ± 0.007
Crystalline IND	125	0.072 ± 0.004
Quenched-in IND	–	0.043 ± 0.004

**Figure 7:**
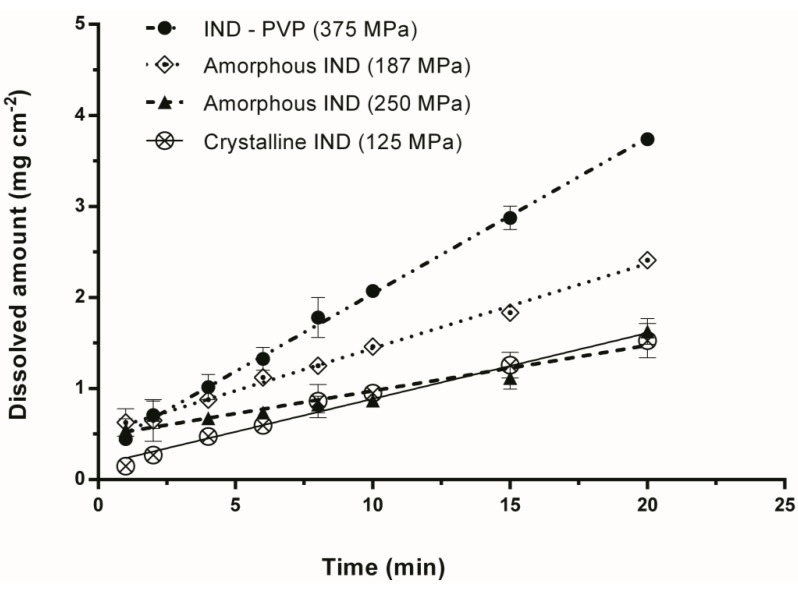
Comparison of dissolution profiles for IND–PVP (375 MPa), amorphous IND (187 MPa), amorphous IND (250 MPa) and crystalline IND (125 MPa) compacts.

Figure 7 illustrates the dissolution profiles of IND–PVP, amorphous IND (milled) and crystalline IND compacts compressed by different compression pressure. It becomes apparent that at a compression pressure of 250 MPa the IDR of amorphous IND is nearly identical to the IDR of crystalline IND, compacted at a compression pressure of 125 MPa. In such a case, the apparent dissolution advantage that the amorphous form possesses could be disregarded. In general, it is suggested that with powder compacts, the IDR is overestimated because of slight changes to the surface during dissolution, whereas the sintered compact showed a more realistic and true value of IDR. However, such sintered compacts could not be obtained for crystalline IND and IND–PVP. Overall, it is suggested that for a reasonable interpretation of the IDR, the surface properties of the different samples need to be taken into account. For amorphous samples in general, the IDR should be measured as a function of the compression pressure.

## 4. Conclusions

This study showed that compression pressure can influence the IDR of pure amorphous IND, whereas crystalline IND and a glass solution of IND–PVP were only marginally influenced by different compression pressure. This could be related to differences in surface properties caused by the compaction. No differences in surface properties were observed for the different IND–PVP compacts and all of them presented almost the same intrinsic dissolution profile. On the other hand, surface differences were observed for the amorphous IND compacts with increasing compression pressure. At low compression pressure, the amorphous IND compacts were powder compacts similar to those of IND–PVP. Above a compression pressure of 250 MPa, however, the amorphous particles sintered to form a single compact with IDR similar to quenched-in disc and crystalline IND compacts. Therefore, the IDR of a sintered amorphous compact might be underestimated when compared to powder compacts of the crystalline substance regardless of the apparent dissolution advantage of the amorphous form.

## References

[1] Khan K.A., Rhodes C. (1976). Effect of variation in compaction force on properties of six direct compression tablet formulations. J. Pharm. Sci..

[2] Iranloye T.A., Parrott E.L. (1978). Effects of compression force, particle size, and lubricants on dissolution rate. J. Pharm. Sci..

[3] Velasco M., Ford J.L., Rowe P., Rajabi-Siahboomi A.R. (1999). Influence of drug: Hydroxypropylmethylcellulose ratio, drug and polymer particle size and compression force on the release of diclofenac sodium from hpmc tablets. J. Control. Release.

[4] Tenho M., Heinänen P., Tanninen V.P., Lehto V.-P. (2007). Does the preferred orientation of crystallites in tablets affect the intrinsic dissolution?. J. Pharm. Biomed. Anal..

[5] Lipinski C.A. (2000). Drug-like properties and the causes of poor solubility and poor permeability. J. Pharmacol. Toxicol. Methods.

[6] Hulse W.L., Gray J., Forbes R.T. (2012). A discriminatory intrinsic dissolution study using UV area imaging analysis to gain additional insights into the dissolution behaviour of active pharmaceutical ingredients. Int. J. Pharm..

[7] Kawabata Y., Wada K., Nakatani M., Yamada S., Onoue S. (2011). Formulation design for poorly water-soluble drugs based on biopharmaceutics classification system: Basic approaches and practical applications. Int. J. Pharm..

[8] Aaltonen J., Rades T. (2009). Commentary: Towards physicorelevant dissolution. Dissolut. Technol..

[9] Anjana M., Joseph J., Nair S.C. (2013). Solubility enhancement methods—A promising technology for poorly water soluble drugs. Int. J. Pharm. Sci. Rev. Res..

[10] Morris K.R., Griesser U.J., Eckhardt C.J., Stowell J.G. (2001). Theoretical approaches to physical transformations of active pharmaceutical ingredients during manufacturing processes. Adv. Drug Deliv. Rev..

[11] Karmwar P., Graeser K., Gordon K.C., Strachan C.J., Rades T. (2012). Effect of different preparation methods on the dissolution behaviour of amorphous indomethacin. Eur. J. Pharm. Biopharm..

[12] Nagapudi K., Jona J. (2008). Amorphous active pharmaceutical ingredients in preclinical studies: Preparation, characterization, and formulation. Curr. Bioact. Comp..

[13] Hilden L.R., Morris K.R. (2004). Physics of amorphous solids. J. Pharm. Sci..

[14] Van den Mooter G. (2012). The use of amorphous solid dispersions: A formulation strategy to overcome poor solubility and dissolution rate. Drug Discov. Today Technol..

[15] Aso Y., Yoshioka S., Kojima S. (2004). Molecular mobility-based estimation of the crystallization rates of amorphous nifedipine and phenobarbital in poly(vinylpyrrolidone) solid dispersions. J. Pharm. Sci..

[16] Yu L. (2001). Amorphous pharmaceutical solids: Preparation, characterization and stabilization. Adv. Drug Deliv. Rev..

[17] Hancock B.C., Parks M. (2000). What is the true solubility advantage for amorphous pharmaceuticals?. Pharm. Res..

[18] Worku Z.A., Aarts J., van den Mooter G. (2014). Influence of compression forces on the structural stability of naproxen/PVP–VA 64 solid dispersions. Mol. Pharm..

[19] Ayenew Z., Paudel A., Rombaut P., van den Mooter G. (2012). Effect of compression on non-isothermal crystallization behaviour of amorphous indomethacin. Pharm. Res..

[20] Greco K., Bogner R. (2010). Crystallization of amorphous indomethacin during dissolution: Effect of processing and annealing. Mol. Pharm..

[21] Nanjwade B.K. (2010). Effect of compression pressure on dissolution and solid state characterization of cefuroxime axetil. J. Anal. Bioanal. Tech..

[22] Wlodarski K., Sawicki W., Paluch K., Tajber L., Grembecka M., Hawelek L., Wojnarowska Z., Grzybowska K., Talik E., Paluch M. (2014). The influence of amorphization methods on the apparent solubility and dissolution rate of tadalafil. Eur. J. Pharm. Sci..

[23] Hendriksen B., Williams J. (1991). Characterization of calcium fenoprofen 2. Dissolution from formulated tablets and compressed rotating discs. Int. J. Pharm..

[24] Lim R.T.Y., Ng W.K., Widjaja E., Tan R.B. (2013). Comparison of the physical stability and physicochemical properties of amorphous indomethacin prepared by co-milling and supercritical anti-solvent co-precipitation. J. Supercrit. Fluids.

[25] Okumura T., Ishida M., Takayama K., Otsuka M. (2006). Polymorphic transformation of indomethacin under high pressures. J. Pharm. Sci..

[26] Savolainen M., Heinz A., Strachan C., Gordon K.C., Yliruusi J., Rades T., Sandler N. (2007). Screening for differences in the amorphous state of indomethacin using multivariate visualization. Eur. J. Pharm. Sci..

[27] Perez-Marcos B., Iglesias R., Gomez-Amoza J., Martinez-Pacheco R., Souto C., Concheiro A. (1991). Mechanical and drug-release properties of atenolol-carbomer hydrophilic matrix tablets. J. Control. Release.

[28] Yadav V., Yadav A. (2009). Enhancement of solubility and dissolution rate of indomethacin with different polymers by compaction process. Int. J. ChemTech Res..

[29] Guilbaud J.B., Cummings L., Khimyak Y.Z. (2007). Encapsulation of indomethacin in PVP: Solid-state NMR studies. Macromol. Symp..

[30] Matsumoto T., Zografi G. (1999). Physical properties of solid molecular dispersions of indomethacin with poly(vinylpyrrolidone) and poly(vinylpyrrolidone-co-vinyl-acetate) in relation to indomethacin crystallization. Pharm. Res..

